# Native soil amendments combined with commercial arbuscular mycorrhizal fungi increase biomass of *Panicum amarum*

**DOI:** 10.1038/s41598-021-97307-2

**Published:** 2021-09-09

**Authors:** Noah C. Luecke, Austin J. Mejia, Kerri M. Crawford

**Affiliations:** 1 Department of Biology and Biochemistry, University of Houston, Houston, TX 77204 USA; 2 Present Address: Department of Entomology, Center for Infectious Disease Dynamics, The Pennsylvania State University, University Park, PA 16802 USA

**Keywords:** Ecology, Microbiology

## Abstract

Coastal dune restorations often fail because of poorly performing plants. The addition of beneficial microbes can improve plant performance, though it is unclear if the source of microbes matters. Here, we tested how native soil amendments and commercially available arbuscular mycorrhizal (AM) fungi influenced performance of *Panicum amarum*, a dominant grass on Texas coastal dunes. In a greenhouse experiment, we manipulated the identity of native soil amendments (from *P. amarum*, *Uniola paniculata*, or unvegetated areas), the presence of soil microbes in the native soil amendments (live or sterile), and the presence of the commercial AM fungi (present or absent). Native soils from vegetated areas contained 149% more AM fungal spores than unvegetated areas. The commercial AM fungi, when combined with previously vegetated native soils, increased aboveground biomass of *P. amarum* by 26%. Effects on belowground biomass were weaker, although the addition of any microbes decreased the root:shoot ratio. The origin of native soil amendments can influence restoration outcomes. In this case soil from areas with vegetation outperformed soil from areas without vegetation. Combining native soils with commercial AM fungi may provide a strategy for increasing plant performance while also maintaining other ecosystem functions provided by native microbes.

## Introduction

Ecological restoration holds promise for recovering degraded ecosystems^1–3^, but restorations are not always successful^4,5^. Many revegetation projects may suffer from low biomass production. Plant performance can be tightly linked to soil conditions, including nutrients and soil microbial community composition^6,7^. Native soils contain microbial communities that can help alleviate plant stress. For example, native microbes can facilitate the acquisition of previously unreachable essential resources^8,9^. Degraded ecosystems often have reduced or altered soil microbial diversity^10–12^, and changes in microbial community composition can produce legacies that impede restoration efforts^13^. Therefore, the successful restoration of native plants may depend on the restoration of soil microbial communities^10,14,15^.

One potential solution for restoring soil communities is the addition of native soil amendments^8,15,16^. Previous work has found that soil amendments, or field soil containing indigenous microbial communities, can influence plant community dynamics. Microbes in native soil amendments from different ecosystems can steer plant community development towards different target communities^17^, and native microbes can defend communities against invasive species^18^. Native microbes can also promote succession in restored communities^19^. However, native soil amendments have yielded mixed results for plant biomass, increasing biomass in some cases^14^ and decreasing biomass in others^20^.

Differences in plant responses to soil amendments may be caused by differences in microbial community composition. In addition to mutualists, including nitrogen-fixing bacteria and arbuscular mycorrhizal (AM) fungi, soils also contain decomposers that can increase soil nutrient quantity and pathogens that decrease plant performance. Soil microbial community composition varies depending on plant species identity^21^. AM fungi often associate with many plant species^22^, while pathogens frequently have host specific effects^23^. The accumulation of host-specific pathogens in native soils is common^24^ and can decrease plant performance^25^. While host-specific pathogens may play an important role in promoting plant diversity^23,24^, they may be detrimental to the early stages of restoration, especially if only one or two species is planted. Therefore, it may be important to consider plant identity when collecting soil amendments for restoration, because there may be a build-up of species-specific pathogens that makes it unadvisable to collect soil near the species that is being restored.

As an alternative to using native soil amendments to introduce soil microbes, commercial AM fungi can be added to restorations. Using commercial AM fungi can increase plant performance^16^ while avoiding the negative effects of native pathogens. However, commercial AM fungi may not provide the same benefits as locally-adapted mutualists^26,27^. Many commercial mixes likely contain early successional fungi that may act more like parasites than mutualists^26^. To combat this, suppliers are producing more diverse mixes of AM fungi derived from native ecosystems that may provide greater and more tailored benefits to plants. When remnant prairie AM fungi communities containing higher diversity were added to prairie restorations, plant biomass and ecosystem functions increased compared to lower diversity treatments^28,29^.When added to prairie restorations, more diverse AM fungi from remnant prairies yielded greater plant performance as well as increasing other ecosystem functions when compared to treatments^28,29^. However, it is unclear whether the benefits from these AM fungi extend to ecosystems other than native tallgrass prairies.

If added together, commercial AM fungi will likely interact with native soil microbial communities to influence plant performance. The addition of commercial AM fungi can significantly alter native soil community composition^30^. Such alterations may lead to greater plant productivity if they increase the diversity of plant mutualists. For instance, increased AM fungal species richness can increase plant productivity^31^. However, whether microbial diversity positively influences plant productivity can depend on the identity of the microbes^32^, so it is important to document how increased diversity resulting from the addition of both commercial and native microbes influences plants. AM fungi can also protect plants from soil pathogens^33^. Therefore, adding commercial AM fungi with native soil amendments may help increase plant performance by decreasing the effect of pathogens that may be present in native soils while increasing the possibility of including necessary locally-adapted microbiota.

Texas Gulf Coast dunes border the Gulf of Mexico and extend into coastal tallgrass prairies. In addition to providing habitat for insects, birds and mammals, these dune systems protect inland ecosystems from wind and waves during coastal storms^34^. Dunes also protect human developments from storm disturbance resulting in a high economic value. With forecasted sea level rise and the increased intensity and frequency of storms, dune stabilization has become a primary focus for restoration practitioners^3^. Revegetation is often essential for the stabilization of coastal foredunes^35–39^. *Panicum amarum* is a dominant grass species that helps stabilize dunes with its above- and below-ground biomass, and it is often used in restoration. However, restorations with *P. amarum* sometimes suffer from high plant mortality and erosion^37^. *Panicum amarum* associates with AM fungi^15^.

In this study, we tested how native soil amendments and a diverse commercial AM fungi mix derived from native prairies influenced the performance of a dominant grass species, *Panicum amarum* (Elliott), that is commonly used in Gulf Coast dune restorations. Dune soils are often nutrient-poor, and both soil organic matter and nutrients can be limited to areas around plants^40,41^. Dune restorations along the Gulf Coast of Texas, USA, are often conducted in areas with no plants, and AM fungi as well as other plant associated microbes are rarer in unvegetated dunes compared to vegetated dunes^10,16^. We hypothesized that (i) the effect of native soil amendments on *P. amarum* performance will depend on soil identity (i.e., whether the soil was collected underneath a conspecific, heterospecific, or no plant), and (ii) the addition of commercial AM fungi will interact with native soil amendments to increase growth of *Panicum amarum*.

## Methods and materials

To test how native and commercial soil microbes influenced the performance of *Panicum amarum* we established a fully factorial greenhouse experiment where we manipulated the identity of native soil amendments (from *P. amarum*, *Uniola paniculate* (Linnaeus), or unvegetated areas), the presence of soil microbes in the native soil amendments (live or sterile; to tease apart effects of differences in soil communities and abiotic soil properties), and the presence of commercial AM fungi (present or absent).

### Collection of native soil amendments

We collected soil for the native soil amendments from vegetated dunes on Galveston Island, TX. All research complied with institutional, local, and national guidelines for the collection and research use of plant and soil microbes. To obtain a wide range of soil communities, we selected three sites that were approximately 3 km apart. At each site, we collected eight replicates of each soil with three different “soil identities”: the rooting zone of *Panicum amarum*, the rooting zone of *Uniola paniculata* (another common dune grass), and bare ground that was free of vegetation for at least one meter. Replicate soil samples were collected at least three meters apart from one another and were kept separate to avoid pseudoreplication. Prior to planting, the soil was stored in a 4 °C cold room to slow microbial processes.

### Plant propagation

To maintain similarity to restoration practices and maintain control of the plants life history we chose to propagate plants from seed. Seeds of *P. amarum* were acquired from Roundstone Native Seed (Uptown, KY). Prior to planting, we surface sterilized the seeds in a 0.08% hypochlorite solution for 10 min and rinsed them with deionized water. Seeds were planted in sterilized play sand (Quikcrete Atlanta, GA) and watered every 2–3 days. Play sand was sterilized by autoclaving twice at 121 °C for one hour, with 24 h in between cycles﻿.

### Experiment

Upon emergence of the first true leaf, *P. amarum* seedlings were transferred into 262 mL conical pots (5 cm diameter × 17.8 cm depth; Stuewe & Sons, Tangent, OR) filled with 225 mL of sterilized play sand. Pots were lined with paper towels to stop sand from draining out of the pots but allow water to pass through. When transplanting the seedlings, we added 25 ml of native soil amendments to the root zone from one of the three soil identities (from *P. amarum*, *U. paniculata*, or unvegetated areas) that was either live or sterile (presence or absence of native soil microbes). We sterilized the native soil amendments by autoclaving at 121 °C for one hour, twice, with 24 h in between cycles. We added 5 g of commercial AM fungi (MycoBloom, Lawrence, KS) to half of the pots in each native soil amendment identity × native microbe presence combination. MycoBloom was developed to preserve the natural diversity of AM fungal communities found in remnant Midwest prairies; therefore, we expect that this commercial AM fungi is more likely to have positive effects on native (non-agricultural) plant performance than other commercial AM fungi which are targeted to non-C4 plant species. MycoBloom contains *Claroideoglomus claroideum* (Schenck & Smith)*, Funneliformis mosseae* (Nicolson & Gerdemann)*, Cetraspora pellucida* (Nicolson & Schenck)*, Claroideoglomus lamellosum* (Dalpé, Koske & Tews)*, Acaulospora spinosa* (Walker & Trappe)*, Racocetra fulgida* (Koske & Walker) and *Entrophospora infrequens* (Hall) (stored in calcined clay. Based on a previous experiment we saw no significant difference in *P. amarum* performance between plants inoculated with sterilized MycoBloom and sterile play sand suggesting the calcined clay alone provides no significant advantage to *P. amarum’s* growth (F_1,52_ = 0.18, P = 0.93)(Luecke unpublished). Each treatment combination was replicated 8 times for a total of 288 pots [3 native soil amendment identities × 2 native soil amendment microbe treatments (live, sterile) × 2 AM fungi treatments (present, absent) × 3 native microbe collection sites × 8 replicates]. Plants were grown in ambient temperatures at the University of Houston greenhouse from May to September 2017 to represent *P. amarum*’s natural growing season. Temperatures ranged from 25–35 °C and relative humidity was ~ 80%. Plants were provided 100 ml of water twice a week. After two months of growth, we fertilized each pot with 100 mL of half-strength Hoagland’s nutrient solution without phosphorus^42^. At the end of the experiment (93 days), we harvested aboveground and belowground plant biomass and dried the biomass at 60 °C for four days prior to weighing.

### AM fungal spore extraction

To determine whether native soil amendment identity influenced the abundance of AM fungi available to colonize plants, we extracted and counted AM fungal spores. Following protocols developed by the International Culture Collection of (Vesicular) Arbuscular Mycorrhizal Fungi (invam.wvu.edu), we extracted spores from each replicate of native soil amendment identity at each site (3 native soil amendment identities × 3 sites × 8 replicates = 72 samples). For each sample, we blended 25 ml of soil with 250 ml of water and poured the suspended particles through a stack of sieves, 1 mm upper and 38 µm lower. The catchments from the lower sieve were collected in a 50 ml tube and centrifuged at 2000 rpm for 2 min with a 60% sucrose solution. The supernatant, containing spores and hyphae, was poured through a 38 µm sieve and rinsed with deionized water. Spores were transferred to a petri dish and quantified at 100 × magnification.

### Statistical analyses

We tested the effects of our treatments using ANOVAs with the response variables of aboveground plant biomass, belowground plant biomass, total plant biomass, and root:shoot ratio. We used ANOVAs with the factor site, followed by native soil amendment identity, native microbe presence, commercial AM fungal presence, and all possible interactions. Site was excluded from interactions as we were interested only in its main effect. Analysis of variance was conducted using type I (sequential) sum of squares. The order in which we analyzed the variables did not qualitatively change the results. Root:shoot ratio was log-transformed to improve normality. Samples that died during the experiment were removed from the analysis of root:shoot ratio. We tested how native soil amendment identity influenced the abundance of native AM fungal spores using ANOVA (type I sum of squares) with the main effects of native soil amendment identity and site. Changing the order in which the main effects were analyzed did not qualitatively effect the results. Spore abundance was log-transformed to improve normality. All statistics were conducted in the base R packages^43^.

## Results

**Figure 1:**
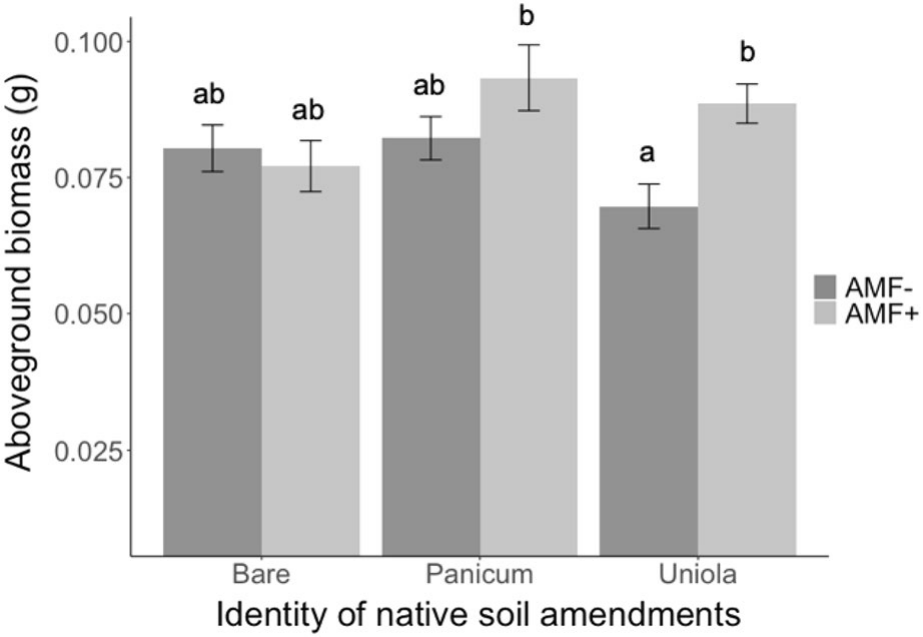
Effect of native soil amendment identity and the presence of commercial AM fungi (AMF−, AMF +) on the aboveground biomass of *P. amarum.* Bars indicate treatment means ± SE. Letters denote significant differences between each combination of soil amendment identity and AM fungi treatment estimated using linear models (P < 0.05).

Across all treatments, the presence of commercial AM fungi increased the aboveground biomass of *P. amarum* by 13.6% (F_1,274_ = 5.21, P = 0.02, Table 1), but the degree of increase depended on the identity of the native soil amendment (F_2,274_ = 4.78, P = 0.01, Table 1, Fig. 1). When commercial AM fungi were added to soils from *U. paniculata* aboveground biomass of *P. amarum* increased by 35.5%. However, commercial AM fungi did not cause a significant increase in aboveground biomass when native soil amendments came from bare ground. The interaction between commercial AM fungi and the identity of the native soil amendment produced qualitatively similar results for total biomass, although results from the post hoc test revealed no significant differences (Tukey’s HSD P = 0.30) (Fig. S1). The effect of the native soil amendments on aboveground and total biomass was not dependent on the presence of native microbes in the soils (i.e., no significant main or interactive effect of native soil microbe presence) (aboveground F_2,274_ = 0.40, P = 0.67 and total F_2,274_ = 0.53, P = 0.59).

**Table 1:** Results from linear models testing the effects of the identity of native soil amendments (Soil ID), the presence of commercial AM fungi (AM Fungi), and the presence of native microbes on aboveground biomass, belowground biomass, root:shoot ratio, and total biomass of *P. amarum*.

Treatment	*df*	Aboveground biomass		Belowground biomass		Root:shoot		Total biomass	
		*F*	*P*	*F*	*P*	*F*	*P*	*F*	*P*
Site	2	0.85	0.43	0.78	0.46	0.45	0.64	0.86	0.42
Soil ID	2	0.64	0.53	0.82	0.44	4.62	**0.01**	0.30	0.74
AM Fungi	1	5.21	**0.02**	0.33	0.56	19.39	** < 0.001**	0.57	0.45
Native microbes	1	0.43	0.51	0.20	0.66	2.81	*0.10*	0.00	0.96
Soil ID × AMF	2	4.78	**0.01**	2.34	*0.10*	1.90	0.15	3.65	**0.03**
Soil ID × Native microbes	2	0.40	0.67	0.58	0.56	0.10	0.90	0.53	0.59
AMF × Native microbes	2	0.89	0.35	3.75	**0.05**	4.08	**0.04**	2.44	0.12
Soil ID × AMF × Native microbes	2	0.40	0.67	0.15	0.86	0.19	0.83	0.27	0.77
Error	274					*df* = 255			

Bolded numbers are significant at *P* < 0.05. Italicized numbers are marginally significant at *P* < 0.10.

**Figure 2:**
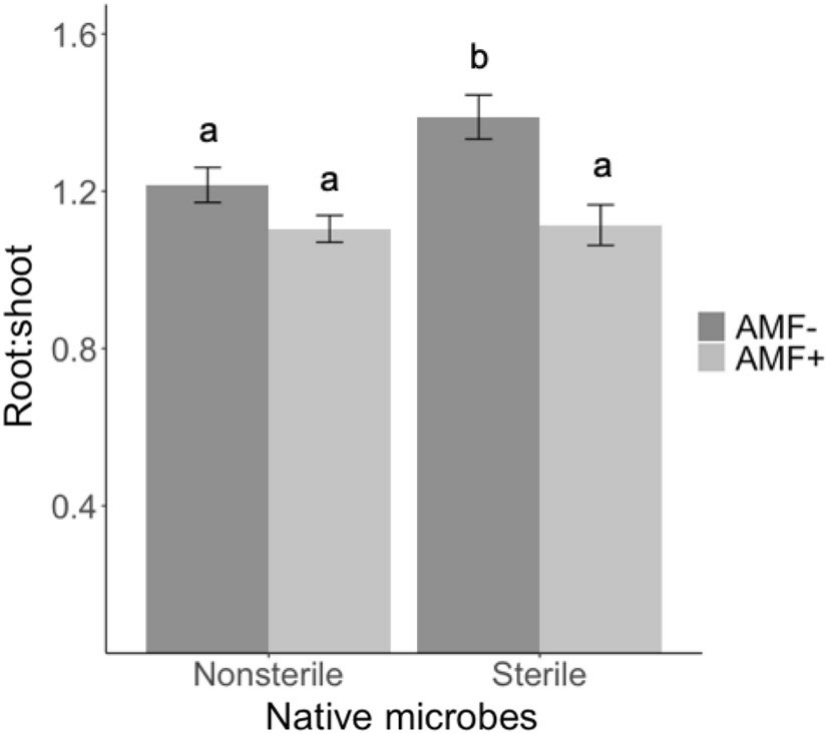
Effect of the presence of native soil microbes and AM fungi on the root:shoot ratio of *P. amarum*. Bars indicate treatment means ± SE. Letters denote significant differences between each combination of presence of native soil microbes and AM fungi treatment estimated using linear models (P < 0.05).

**Figure 3:**
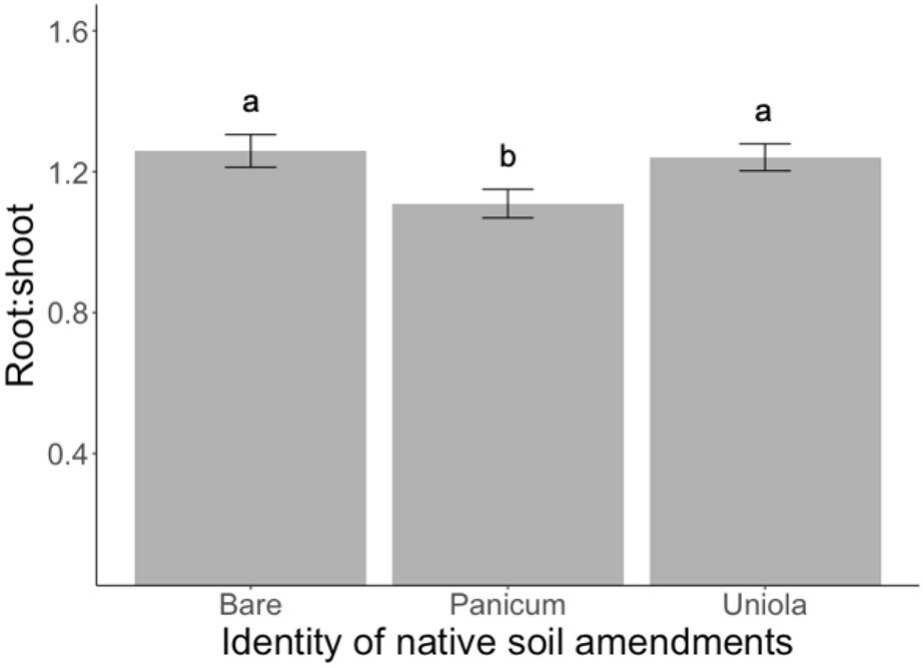
Effect of the identity of native soil amendments on the root:shoot ratio for *P. amarum.* Bars indicate treatment means ± SE. Letters denote significant differences between native soil amendments estimated using linear models (P < 0.05).

The presence of native microbes did interact with commercial AM fungi to influence *P. amarum* belowground biomass (F_2,274_ = 3.75, P = 0.05) and root:shoot ratio (F_2,255_ = 4.08, P = 0.04, Table 1). There was a trend for commercial AM fungi to decrease belowground biomass for plants with no native microbes by 15.2% (Fig. S2); however, the posthoc test revealed no significant difference (Tukey’s HSD: P = 0.29). In the absence of native soil microbes, the addition of commercial AM fungi significantly decreased root:shoot by 19.4% (F_2,255_ = 4.08, P = 0.04, Table 1, Fig. 2). In fact, the presence of any microbial addition (AM fungi or native soil microbes) significantly decreased the root:shoot ratio by an average of 17.5% relative to the sterile native soil, no commercial AM fungi control (F_2,255_ = 4.08, P = 0.04, Table 1, Fig. 2). The identity of native soil amendments also influenced root:shoot ratio (F_2,274_ = 4.62, P = 0.01, Table 1). Native soil amendments from *P. amarum* produced a lower root:shoot ratio than amendments from bare ground or *U. paniculata* (Fig. 3).

**Figure 4:**
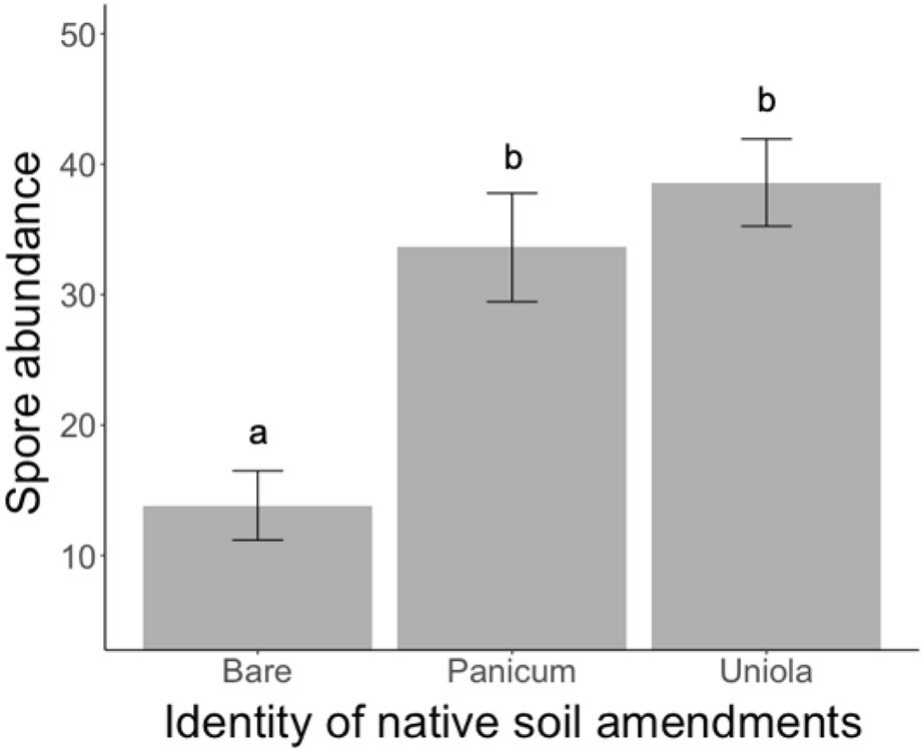
Effect of native soil amendment identity on the abundance of arbuscular mycorrhizal fungal spores. Spores were extracted from 25 ml of soil. Bars indicate treatment means ± SE. Letters denote significant differences between native soil amendments estimated using linear mixed-effects models (P < 0.05).

The abundance of native AM fungal spores varied significantly based on native soil amendment identity (*F*_1,71_ = 11.18, *P* < 0.0001). Bare soil contained 63.5% fewer spores than soils from *U. paniculata* and 55.2% fewer spores than soils from *P. amarum* (Fig. 4). There was no correlation between spore abundance and plant biomass (aboveground *F*_1,65_ = 0.23, *P* = 0.62; belowground *F*_1,65_ = 0.02, *P* = 0.90; total *F*_1,65_ = 0.06, *P* = 0.80).

## Discussion

The use of native soil amendments in combination with commercially available AM fungi derived from a native prairie significantly influenced the performance of the Gulf Coast dune grass, *Panicum amarum*. The commercial AM fungi increased aboveground biomass for *P. amarum,* but only when it was grown with native soil collected from the native dune grasses *P. amarum* or *U. paniculata*. Changes in root:shoot ratio also occurred in our treatments, with plants containing a higher proportion of roots in the absence of native or commercial microbes. Belowground biomass, however, was relatively unresponsive to our treatments – although there was a trend for the commercial AM fungi to decrease belowground biomass in the absence of live native microbes. In sum, the use of live native soil from vegetated areas in combination with a diverse commercial AM fungi mix derived from native prairie increased aboveground biomass without significantly changing belowground biomass, while also increasing the abundance of native AM fungal spores.

Our results indicate that soil origin is important to consider when using native soil amendments in restorations. We found that in order for native soil amendments to enhance the effects of the commercial AM fungi on aboveground biomass, the soil amendments had to come from vegetated areas. Contrary to our predictions, this effect was not driven by the presence of native soil microbes. This is particularly surprising as P. amarum has many mycorrhizal-dependent congeners. Therefore, some other property of the native soils was driving the observed response. We suspect that nutrient availability was greater in soils from vegetated areas than unvegetated areas, as has been found in other studies^44^. Even though we added in a relatively small amount of the native soil amendments, a small increase in soil nutrients may have had a big impact on plant performance in the nutrient-poor sandy background soil. If the native vegetated soils did increase nutrient availability, they may have provided a greater pool of nutrients for commercial AM fungi to access. The identity of the native soil amendments also influenced the root:shoot ratio of *P. amarum*, with the lowest ratio in soils from *P. amarum.* While it is unclear what is driving this relationship, it is possible that soils from conspecifics have a greater availability of nutrients needed by *P. amarum*, reducing the need for the plants to produce more roots to seek out nutrients. The role of soil nutrients in driving *P. amarum* performance could be resolved by future work that measures nutrient availability in conspecific, heterospecific, and unvegetated soils followed by tests of how the addition of certain nutrients influence plant performance. This could lead to the development of fertilizers designed to increase plant performance in dune restorations*.*

In contrast to native soil amendments, commercial microbes are more commonly used in restoration projects. Commercial microbes are easily obtained and, unlike native soils, unlikely to contain soil enemies that may decrease plant performance. However, there are concerns that commercial microbes may not provide the same benefits as native microbes that are locally adapted to site-specific conditions^27,29^. Our results demonstrate a case where commercial microbes increased plant performance more than native microbial inocula. However, this may not be true for all commercial inocula. Many commercial suppliers of AM fungal innoculum focus on fungi that are selected to improve agricultural crop performance. In contrast, the commercial AM fungi we used was developed from native prairies. This too may have generated a mismatch between fungi and plant-host as *P. amarum* is a coastal dune species. Our findings raise the question of how local native microbes need to be in order to provide the greatest benefits to restorations. This may be especially true as species overlap may occur between environments but local adaptation and life history may alter plant–microbe interactions. While our experiment was not designed to answer this question, we found that microbial communities from native prairies outperformed locally derived dune microbial communities. Future work that explicitly tests for local adaptation in microbial communities and documents a wider range of responses would greatly inform the management of microbial communities in restorations^45^.

There has been growing interest in using native soils, along with their native microbes, to improve restoration outcomes^17,46^. When the efficacy of native soil amendments is in question, our results suggest that bet-hedging by adding both a native commercial AM fungi mix and native soil amendments may be an effective strategy for maximizing plant performance. Even though our live native soil amendments contained AM fungi, the native AM fungi did not provide the same benefits as the commercial AM fungi. Different AM fungi can have different effects on plant performance^47^, so there may have been a difference in composition of AM fungal communities in the native soils and the commercial mix. Another likely explanation for the weak effect of dune-derived AM fungi is that their abundance in native soils may have been too low to elicit immediate strong responses from plants. Alternatively, interactions between the dune-derived AM fungi and native soil pathogens may have produced a neutral effect on plant growth. Finally, not all AM fungi are beneficial to their plant hosts in all situations^48^. These ideas could be tested with experiments that manipulate the composition, quantity, and presence of AM fungi and soil pathogens in trials with *P. amarum*.

Our experiment may underestimate the effects of plant–microbe interactions as they are often context-dependent^8^, and mutualisms may have stronger effects in more stressful environments^48^. Therefore, the positive effects of microbes may be even stronger in the relatively harsh dune environment than they were in the greenhouse. The lack of effects on belowground biomass may also have been limited by pot size, potentially masking any differences between treatments. Furthermore, we were unable to measure many other responses of interest that may be influenced by microbes, including long-term plant performance, plant resistance to and resilience following disturbance, and ecosystem functions such as carbon sequestration and soil development^49^. Future experiments that test the effect of commercial and native microbes on restoration performance in the field are needed. Importantly, while the microbes in native soil amendments only had weak effects on plant performance in our experiment, they may provide critical services that we were unable to measure in our experiment that would be evident in a longer-term field experiment^20^.

Given the importance of plant success for restorations, finding an effective way to increase plant performance is critical. By demonstrating how native soil amendments and a diverse commercial AM fungi mix independently and interactively influenced *P. amarum,* we are able to suggest a strategy for optimizing *P. amarum* growth. Using commercial AM fungal derived from native ecosystems in combination with soils collected from vegetated dunes can help grow more robust plants which may provide greater ecosystem services, including habitat for other species and buffering of inland areas from the sea during large storm events. Importantly, only small amounts of native soil amendments are needed to gain this benefit (less than 25 ml per plant), so the disturbance caused by collecting soils from intact dunes need not be widespread. We found no evidence of native dune microbes negatively affecting *P. amarum,* indicating no need for sterilization of native soil amendments. Furthermore, native microbes can contribute to other important to ecosystem functions, such as improving carbon and nitrogen cycling, stabilizing soil, and promoting plant diversity^20,50^. Therefore, restoration strategies that combine commercial mutualists with native soils have the potential to improve multiple restoration outcomes^51^.

## Supplementary Information

Supplementary Information.
