# Supplementary material for: Native soil amendments combined with commercial arbuscular mycorrhizal fungi increase biomass of Panicum amarum
Source: Sci Rep. 2021 Sep 9;11:17865. doi: 10.1038/s41598-021-97307-2 (PMC8429433; doi:10.1038/s41598-021-97307-2)
Supplement: Supplementary file 1 — Supplementary Information. [file 41598_2021_97307_MOESM1_ESM.docx]

**Figure S1.** Effect of native soil amendment identity and addition of commercial arbuscular mycorrhizal fungi on total biomass. Measurements are averaged across all treatments. Bars indicate treatment means ± SE. Letters denote significant differences between each combination of identity of native soil amendments and AM fungi treatment estimated using linear models (P < 0.05).


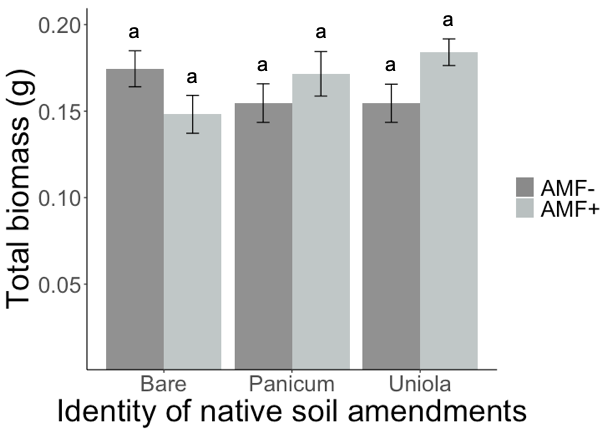


**Figure S2.** Effect of the presence of native soil microbes and AM fungi on the belowground biomass of *P. amarum*. Bars indicate treatment means ± SE. Letters denote significant differences between each combination of presence of native soil microbes and AM fungi treatment estimated using linear models (P < 0.05).

**
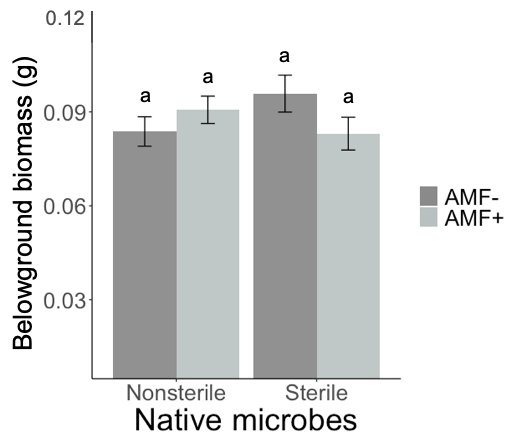
**
